# Rebalancing the Vestibular System by Unidirectional Rotations in Patients With Chronic Vestibular Dysfunction

**DOI:** 10.3389/fneur.2018.01196

**Published:** 2019-01-22

**Authors:** Navid G. Sadeghi, Bardia Sabetazad, Nayer Rassaian, Soroush G. Sadeghi

**Affiliations:** ^1^Department of Physiology, Shahid Beheshti University of Medical Sciences and Health Services, Tehran, Iran; ^2^Audiology and Dizziness Center, Day General Hospital, Tehran, Iran; ^3^Department of Communicative Disorders and Sciences, Center for Hearing and Deafness, University at Buffalo, Buffalo, NY, United States

**Keywords:** compensation, unidirectional rotation, vestibulo-ocular reflex, directional preponderance, rehabilitation

## Abstract

**Introduction:** Vestibular dysfunction is a common disorder that results in debilitating symptoms. Even after full compensation, the vestibulo-ocular reflex (VOR) could be further improved by using rehabilitation exercises and visual-vestibular adaptation. We hypothesized that in patients with asymmetric vestibular function, the system could be rebalanced by unidirectional rotations toward the weaker side (i.e., a pure vestibular stimulation).

**Methods:** Sixteen subjects (5 female and 11 male, 43.2 ± 17.0 years old) with chronic vestibular dysfunction that was non-responsive to other types of medical treatment were recruited for the study (ClinicalTrials.gov Identifier: NCT01080430). Subjects had VOR asymmetry quantified by an abnormal directional preponderance (DP) with rotation test and no previous history of central vestibular problems or fluctuating peripheral vestibular disorders. They participated either in the short-term study (one session) or the long-term study (7 visits over 5 weeks). Rehabilitation consisted of five trapezoid unidirectional rotations (peak velocity of 320°/s) toward the weaker side. Care was taken to slowly stop the rotation in order to avoid stimulation in the opposite direction during deceleration. To study the short-term effect, VOR responses were measured before and 10, 40, and 70 min after a single unidirectional rotational rehabilitation session. For long-term effects, the VOR gain was measured before and 70min after rehabilitation in each session.

**Results:** We observed a significant decrease in VOR asymmetry even 10 min after one rehabilitation session (short-term study). With consecutive rehabilitation sessions in the long-term study, DP further decreased to reach normal values during the first 2 sessions and only one subjects required further rehabilitation after week 4. This change in DP was due to an increase in responses during rotations toward the weaker side and a decrease in VOR responses during rotations in the other direction.

**Conclusion:** Our results show that unidirectional rotation can reduce the VOR imbalance and asymmetry in patients with previously compensated vestibular dysfunction and could be used as an effective supervised method for vestibular rehabilitation even in patients with longstanding vestibular dysfunction.

## Introduction

Normal vestibular function is essential for proper balance control and gaze stabilization during head movements during natural activities. Vestibular dysfunction results in imbalance between inputs from the two sides, leading to symptoms such as vertigo. Vestibular disorders have a prevalence of ~35% in Americans above 40 years of age (1). The dysfunction has considerable impact on daily activities, requiring sick leaves in ~80% of cases and puts a large burden on health costs. Vestibular system's great adaptive properties are exploited during vestibular compensation, a process that includes changes in the vestibular periphery (2), vestibular nuclei (3, 4), commissural connections between the two nuclei (5) and extravestibular inputs (6–8).

Evidence from previous studies suggest that natural vestibular compensation strategies do not use the full capacity of the system. Training programs that use visual-vestibular training in the form of bidirectional (9) or unidirectional (10) rotations in the presence of a visual surround further improve the vestibulo-ocular reflex (VOR) in animals with compensated unilateral lesions.

In order to improve compensation in patients with chronic vestibular symptoms, the multisensory nature of the vestibular compensation can be exploited through sets of rehabilitation exercises (11–15). Originally, vestibular rehabilitation was performed as group activities and a hierarchy of exercises with different difficulty levels (16). Later, more specific approaches were used based on physiological or behavioral rationales, which were more effective in decreasing the magnitude of symptoms experienced by patients and increasing their independence during daily activities (17, 18). Recently, it has been shown that customized and supervised exercises are more beneficial than unsupervised (e.g., performed alone at home) or general fitness exercises (19–24).

Here, we describe a new rehabilitation method that solely targets the vestibular pathway through a specific vestibular stimulation. The rehabilitation consists of unidirectional rotations in the dark in the direction of the less responsive (LR) side. The hypothesis behind this original idea was formalized by one of the authors (NR) and tested by pilot (unpublished) studies about 20 years ago. Basically, this hypothesis was based on changes in commissural pathways and vestibular nuclei during compensation and suggested that unidirectional rotation toward the side with lower VOR responses results in excitation of that side and simultaneous inhibition of the other side (i.e., the side with higher VOR responses). This could result in an adaptive change, leading to an increase in responses of the weaker side and a new balance between the two sides. This effect could be due to changes in the vestibular nuclei and commissural pathways or at the peripheral level, or both. A confounding and counterproductive effect most likely also exists due to the habituation of responses resulting from repeated rotations, as shown by previous studies in normal animals and humans (25–30). We provide evidence that a pure unidirectional rotation in patients with vestibular asymmetry could effectively reduce the VOR asymmetry, with effects lasting for several weeks. In some, but not all cases, this was accompanied by a long-term subjective sense of improvement in balance.

## Methods

This study was performed as a sequential double blinded clinical trial on 16 patients (5 females and 11 males, 25–64 years old). There was no sex or age limitation for selecting the patients. Regarding the etiology of the vertigo, during our initial assessment, we only asked questions to rule out any known central etiology, such as tumor or surgery (since it would interfere with compensation process) or any history of fluctuating disorders such as Meniere's or BPPV with asymptomatic periods (which would be inappropriate for studying the rehabilitation effects). Typically, subjects' symptoms were not alleviated by previous medical treatment and none of the subjects used any medication during the study. Subjects had a proven and documented history of vestibular dysfunction for 1–8 years and an abnormal asymmetric VOR response during rotation test, as evidenced by a directional preponderance (DP) >10% during rotation (see below). In the initial session, a complete vestibular examination was performed, which included saccadic, smooth pursuit, optokinetic, gaze holding, rotation, and caloric tests. We used caloric DPs as supplementary evidence of asymmetry (DP < 20%) initially. However, caloric DP was not required to be abnormal for inclusion in the study. Caloric DPs were positively correlated with rotation DPs (*R*^2^ = 0.69). The research protocol was then explained to patients and those who agreed to participate in the study, gave written informed consent in accordance with the Declaration of Helsinki. Subjects were free to drop out of the study at any time. All tests were performed in the Audiology Center of Day General Hospital, Tehran, Iran and each patient's primary care physician or otolaryngologist was informed of their participation in this research. This study (ClinicalTrials.gov Identifier: NCT01080430) was carried out in accordance with the recommendations of the Ethics Committee of Shahid Beheshti University of Medical Sciences, Tehran, Iran and the protocol was approved by the Institutional Review Board of the University.

### Quantifying the VOR Asymmetry

Eye movements were measured by electronystagmography during rotation (Nicolet Spirit). Rotations were performed at peak velocity of 40°/s and 0.2 Hz. Patients were in complete darkness with eyes open during the test. The head was positioned 30° nose down, so that the horizontal canal was in its maximum plane of activation. All recordings were done while the subjects performed mental arithmetic to increase their state of alertness.

As a measure of vestibular compensation (10, 31, 32), VOR symmetry was quantified by calculating the directional preponderance (DP) as:

$$\begin{array}{l} DP=\frac{V_{HR}-V_{LR}}{V_{HR}+V_{LR}}\times 100 \end{array}$$

where V_HR_ and V_LR_ represent peak eye velocities during rotations toward the side with higher responses (HR) and lower responses (LR), respectively. The HR and LR sides were determined on the first test for each subject and were not changed during the course of the study. In this way, a change in the direction of DP would be represented by negative values. The normal range of DP for the test as performed by our equipment was <8% as measured in 52 normal subjects. Patients with initial DP values of >10% were included in the study.

We used DP of responses to whole-body sinusoidal rotations as a measure of asymmetry to quantify the effect of our intervention. Comparison of rotation DP to caloric test has shown that it is a reliable measure of diagnosis of vestibular imbalance in routine vestibular clinical practice and follow up (33, 34). It also has the additional benefit that its measurement is fast and relatively comfortable for patients (35). Furthermore, whole body rotation provides reliable results that are comparable to head-on-body rotations during head shaking (36) or head impulse test (HIT) (35, 37). Finally, while both HIT and whole body rotations reliably track ipsilesional VOR recovery, whole body rotations are better for following contralesional compensatory changes over time (37).

### Short-Term and Long-Term Unidirectional Rotational Rehabilitation Protocol

The unidirectional rotation comprised of a velocity trapezoid, with acceleration of 80°/s^2^ over 4 s to reach a maximum velocity of 320°/s and then slowly decelerate at 10°/s^2^ to stop over approximately 30 s. The slow deceleration was particularly important in order to have a smooth end of rotation since a sudden stop could function as a stimulation in the opposite direction. Each session comprised of 5 such rotations, with 1 min intervals in between. The whole session was completed in ~7 min. Rotations were performed in the dark with the subjects' eyes open and heads positioned 30 degrees nose down.

In each session of the study, subjects first underwent an initial DP assessment by rotation test. After 3–4 min, the unidirectional rotational rehabilitation was performed as described above. Eight subjects participated in a short-term study, for which the subjects were kept in the rotation chair and DP was assessed by sinusoidal rotation test 10, 40, and 70 min after the end of rehabilitation. VOR asymmetry was originally evaluated by rotation and caloric tests, but for further evaluations we only used rotational testing since it was less bothersome for patients and more practical for serial evaluations. Another 8 subjects participated in a long-term study. In this case, subjects were asked to rest for 1 h in a calm place in the hospital without using stimulating beverages (e.g., coffee) and post rehabilitation DP was measured only 70 min after the unidirectional rotation. The rehabilitation was performed two times a week for the first 2 weeks and once a week for the second 2 weeks, providing a total of 6 sessions in 4 weeks. One week after the last session, a sinusoidal rotation test was performed for a final DP measurement. During the course of the study, if the DP measured at the beginning of any session was in the normal range or reversed, the patient was not subjected to any additional unidirectional rotations and would be instructed to return for follow up in the next session. We did this as an ethical issue since the unidirectional rotation in a subject with normal DP was not necessary and could theoretically result in an imbalance in the opposite direction.

### Evaluation of Subjective Improvement of Symptoms

To document symptoms of all patients before the beginning of the study in the first session, they were evaluated by one of the researchers (NGS) using a questionnaire. In particular, they were asked to specify when their vestibular symptoms (e.g., vertigo, falling to one side, oscillopsia) have started, whether they had a sensation of rotation (i.e., true vertigo) or imbalance, the frequency and duration of symptoms, any accompanying auditory problems, and any precipitating factors. Subjects that participated in the long-term study were also asked to fill in a form in order to report any occasions of vestibular symptoms and their specificities (e.g., duration, intensity, …) during the days between the rehabilitation sessions.

## Results

We tested the effect of unidirectional rotational rehabilitation on 16 patients (5 female and 11 male) with confirmed chronic vestibular dysfunction for 1–8 years (3.5 ± 2). All patients had a history of some level of auditory problem, with some degree of hearing loss. Mean age of subjects was 43.2 ± 17.0 (range: 25–64) years old. For the short-term study (*n* = 8 subjects, 3 female, 5 male), data was collected at 10, 40, and 70 min after rehabilitation. For the long-term study (*n* = 8 subjects, 2 female, 6 male), data was collected over 6 sessions (4 weeks), before and 70 min after rehabilitation in each session (see Methods for details). None of the patients had jobs or participated in activities that resulted in intense head movements in between sessions and none had performed rehabilitative physical exercises.

### Short-Term Effect of the Unidirectional Rotation

**Figure 1:**
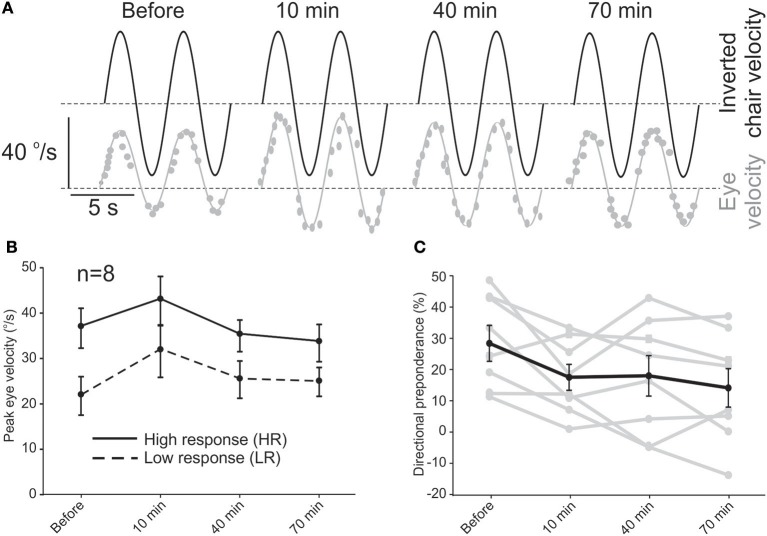
Short-term effect of the unidirectional rotational rehabilitation on VOR asymmetry. **(A)** Example of an asymmetric response to sinusoidal rotation in one of the subjects and its improvement after rehabilitation. Dashed lines show 0°/s. **(B)** Average peak eye velocity (*n* = 8 subjects) for rotations in the two directions before and after rehabilitation. The side that has larger responses is designated as the side with “higher activity” or HA and the other side as “lower activity” or LA. **(C)** Directional preponderance as a measure of VOR asymmetry decreased over time. The change is significant between the initial value and all other values (repeated measure ANOVA, *p* < 0.01). Data for each subject is shown by gray lines.

For each of the 8 subjects that participated in the short-term study, eye velocities were measured during sinusoidal rotations and VOR responses were evaluated for half cycles to the right and left and a DP was calculated. DPs calculated for rotation and caloric tests were linearly related to each other (slope = 0.7) and on average, were not different (26.5 ± 6.5% vs. 30.1 ± 6.0%, paired *t*-test, *p* = 0.36). Based on the initial rotation test (i.e., before rehabilitation), the two sides were labeled as “low response” (LR) and “high response” (HR). The unidirectional rotational rehabilitation was then performed with rotations toward LR as described in the Methods. Figure 1A shows the VOR response for one of the subjects with an initial asymmetric VOR, with smaller responses during rotations to the right. As such, the right side was labeled as LR and rehabilitation for this subject consisted of unidirectional rotations to the right. At 10 min after the end of rehabilitation, there was an increase in responses for rotations in both directions. For this subject, HR responses gradually decreased over time, while LR responses remained slightly larger than initial values.

Average eye velocities for all patients in the short-term study showed a similar trend (Figure 1B). While VOR responses for both sides increased slightly 10 min after rehabilitation, this change was not significant (repeated measures ANOVA, *n* = 8, *p* = 0.08) and decreased at 40 min and 70 min for both directions of rotation. While the increase in eye velocity for LR rotations could be attributed to the unidirectional rotation (i.e., our hypothesis), the increase for HR half cycles at 10 min was unexpected and could be a rebound phenomenon after the inhibition due to the fast unidirectional rotation. The general trend of these changes was in a way that the asymmetry between the two sides decreased over time as calculated by the DP value (Figure 1C). This effect was observed even 10 min after rehabilitation (repeated measure ANOVA, *post hoc* Tukey test, *p* = 0.016 re initial value) and continued up to 70 min (*p* = 0.003). Seventy minutes after rehabilitation, DP was normalized in half of the patients, while the other half showed a decrease in DP. In 2 subjects with DP (post-rotation) to within the normal range, the direction of DP changed (i.e., negative DP) at 40 min and in one of them remained so even at 70 min.

Together, these results suggest an effective improvement in VOR asymmetry up to 70 min after one session of the unidirectional rotational rehabilitation. We next investigated whether this effect could be preserved for longer periods.

### Long-Term Effect of the Unidirectional Rotation

Eight patients (2 female and 6male) participated in the long-term study, which required 7 visits over a period of 5 weeks. Note that these subjects were different from those in the short-term study and have not had any previous experience with the unidirectional rotation. Of these, 2 female subjects only participated in the first session and dropped out of the study for personal reasons. For the other 6 subjects, we measured VOR responses at the beginning of each session and 70 min after the rehabilitation in that session.

**Figure 2:**
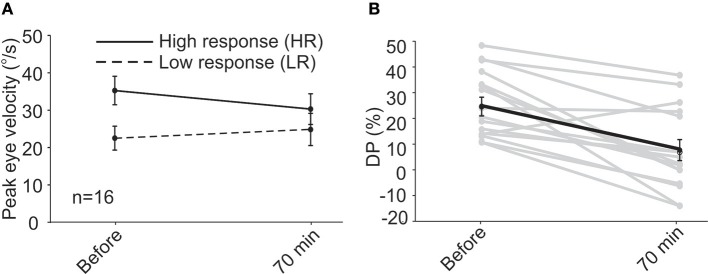
Following unidirectional rotational rehabilitation, VOR response to HA rotations decreased by ~16% and responses to LA rotations increased by ~14% when all 16 patients (short-term and first session of long-term group) were pooled together **(A)**. Although average changes were not significant 70min after rehabilitation, they resulted in a significant change in DP **(B)**, decreasing from 24.7 ± 3.7% to 7.7 ± 4.1% (paired *t*-test, *p* = 0.0006) and bringing it to normal values.

In the first session, similar to that observed for subjects in the short-term study, VOR responses showed a decreasing trend for HR peak eye velocity and an increasing trend for LR peak eye velocity (Figure 2A), the changes were not significant for HR (35.0 ± 3.6 vs. 26.0 ± 4.4°/s, paired *t*-test, *p* = 0.15) or LR (25.0 ± 2.2 vs. 26.75 ± 5.3°/s, paired *t*-test, *p* = 0.23). All of the long-term patients also showed a decrease in their DP values 70 min after rehabilitation in the first session and the average DP decreased from 21.2 ± 4.1% initially to 1.4 ± 4.2% (paired *t*-test, *p* = 0.02). When data of all 16 subjects were pooled together, the decrease at 70 min became more pronounced (Figure 2B, 24.7 ± 3.7% vs. 7.7 ± 4.1%, paired *t*-test, *p* = 0.0006).

**Figure 3:**
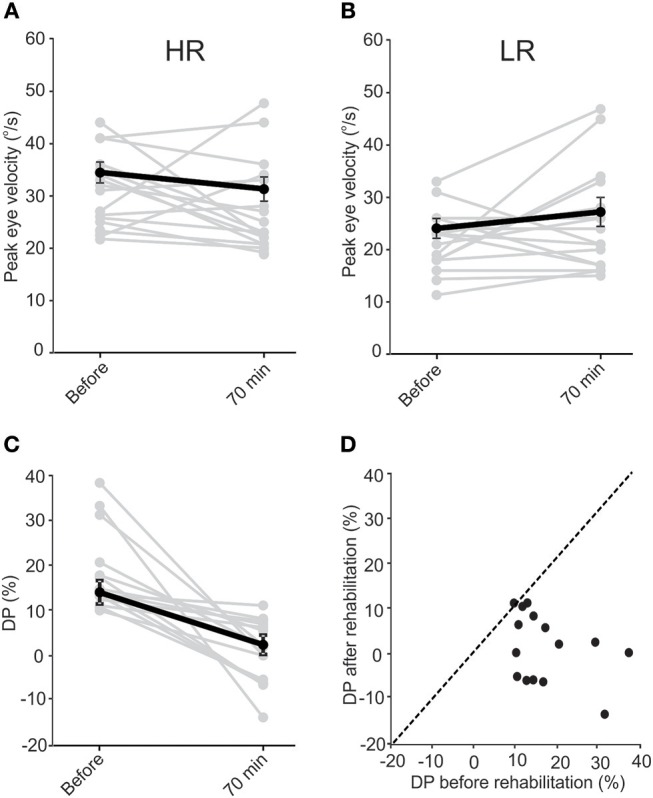
Data pooled from all sessions of subjects in the long-term study. “Before” data is from the initial values collected before rehabilitation in each session. “After” data is collected 70 min after rehabilitation in each session. VOR responses for LA and HA rotations were different “before” rehabilitation (paired *t*-test, *p* = 0.003). Similar to the short-term study, in the majority of instances rehabilitation resulted in a decrease in VOR responses during HA rotations **(A)** and an increase in responses during LA rotations **(B)**, resulting in the responses to rotations in the two directions to be similar after rehabilitation (paired *t*-test, *p* = 0.85). While the change in response averages were not significant for HA (paired *t*-test, *p* = 0.33) and LA (paired *t*-test, *p* = 0.11) responses, the average DP **(C)** still decreased significantly (paired *t*-test, *p* = 0.002). Except in one session for one of the subjects, DPs either decreased or did not change after rehabilitation. This is shown by the points lying below the dashed identity line in **(D)**.

When VOR responses at the beginning of all sessions were pooled (Figures 3A,B), the eye velocities for rotations in the two directions were significantly different (33.8 ± 2.0°/s vs. 23.5 ± 1.5°/s, *t*-test, *p* = 0.003). At 70 min after rehabilitation, the pooled data showed no significant difference between the two sides (31.0 ± 2.3°/s vs. 27.15 ± 2.8°/s). The decreasing (non-significant) trend for HR rotations and the increasing (non-significant) trend for responses to LR rotations were opposite to that expected from simple habituation to a unidirectional rotation observed in normal subjects, which resulted in a decrease in the responses of the side ipsilateral to rotation and no change in the opposite side (38). Differences between responses in normal conditions and in asymmetric (compensated) conditions could be due to compensatory changes in vestibular nuclei neurons and commissural pathways and will be further addressed in the Discussion. As a result of these changes in responses of the two sides, average DP values decreased (Figures 3C,D) from 14.1 ± 2.2% at the beginning of sessions to 2.4 ± 2.2% at 70 min after rehabilitation (paired *t*-test, *p* = 0.002). This change is comparable to that observed for the short-term study (Figure 1C).

**Figure 4:**
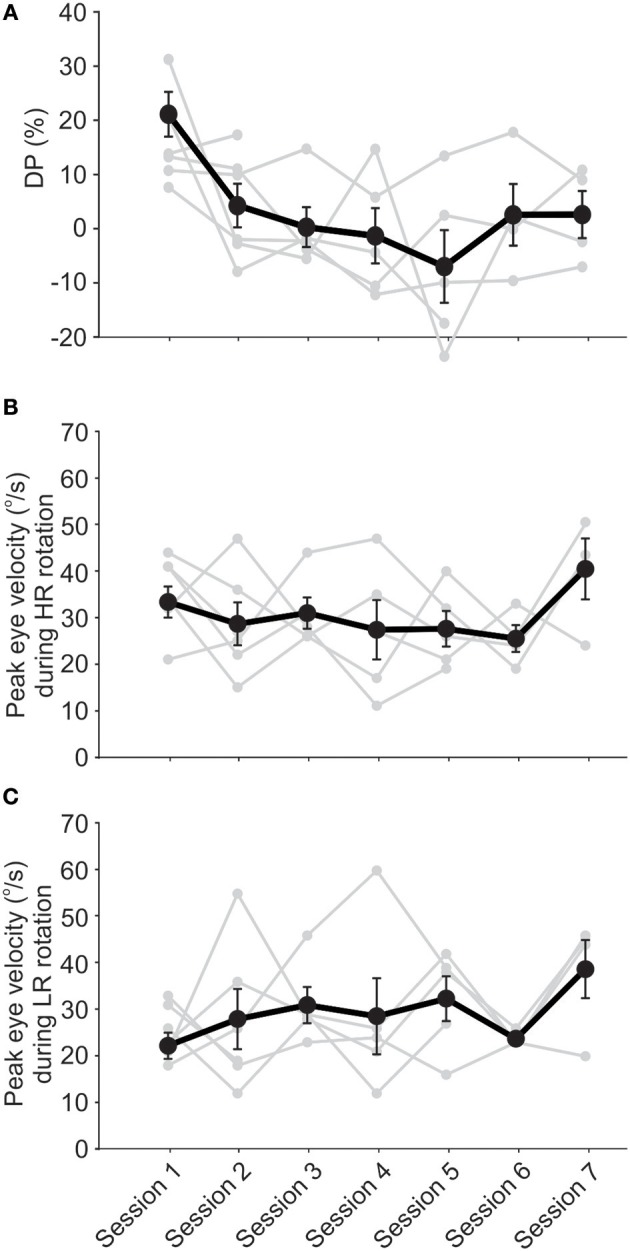
Unidirectional rotational rehabilitation resulted in long-term improvement of VOR asymmetry. Data points are from subjects who participated in at least 2 session (*n* = 6) and the initial values measured before rehabilitation are shown for each session. DP decreased over time for the majority of these subjects **(A)**. The only significant change is between the DP for the first session compared to other sessions (ANOVA, *p* < 0.01), suggesting that the decrease in asymmetry occurs in the first couple of sessions for most subjects. This decrease was due to a decrease in VOR responses to HA rotations **(B)** as well as an increase in responses to LA rotations **(C)**. Changes in VOR responses (i.e., eye velocities or gains) were not significant, but were enough to result in a highly significant improvement in VOR symmetry, as quantified by DP. Note the increase in VOR responses for both sides in the final session, reaching close to normal values (~40°/s).

In the majority of cases, DP decreased to within the normal range in the first few sessions. On average, DP was in the normal range at the beginning of the second session and showed no significant change up to the last session, about 4 weeks later (Figure 4A). Note that when patients showed normal DPs they did not receive rehabilitation and were only followed up in the next session. Similar to the short-term effect, average VOR responses as measured by peak eye velocity did not show a significant change over time (Figures 4B,C, ANOVA, *p* > 0.05). Again, there was a non-significant decreasing trend over time in responses to rotations toward HR and a non-significant increasing trend for responses to LR rotations, which were enough for a significant decrease in asymmetry and DP over time. Also, notice that all 4 subjects who returned for the last final DP measurement (with no rehabilitation rotation) had symmetric VOR responses with minimal DP values. For 3 of these subjects, the symmetry was accompanied by near normal responses (i.e., ~40°/s) for rotations in both directions. Although a significant clinical finding, this should be considered with caution since only 4 subjects participated in the last session and 3 of them had LR peak eye velocities close to normal at this point (Figures 4B,C, last points).

**Figure 5:**
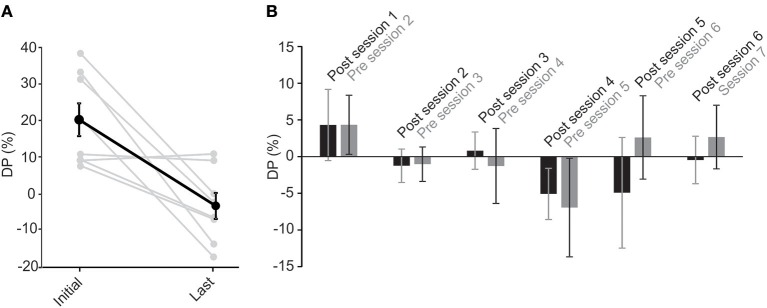
Unidirectional rotational rehabilitation results in long-term effects. **(A)** Comparison of DP values between the initial DP calculated on the first session (i.e., before the first unidirectional rotational rehabilitation) and DP on the last session that a subject participated in the long-term study. Most of the subjects (6 out of 8) showed a decrease in DP after rehabilitation. Although DP in two subjects who had initial near normal DP values, did not change after rehabilitation, average DPs showed a significant decrease (paired *t*-test, *p* = 0.008). **(B)** The effect of rehabilitation was retained in between sessions. Bar graphs show initial values measured at the end of each session 70min after rehabilitation (black, “post session”) and those at the beginning of the next session before rehabilitation (gray, “pre session”). Differences between values were not significant for each group (repeated measures ANOVA, *p* > 0.1 for all), suggesting that the effect of rehabilitation lasted until the next session.

For all subjects, the last recorded DP—either session 7 or the last session that they participated in—was lower than the original value, measured before the rehabilitation on the first session. In fact, all final DPs were within the normal range (Figure 5A). The average DP decreased significantly (paired *t*-test, *p* < 0.05) from 14.8 ± 3.8 to −2.2 ± 4.4. Notably, the rehabilitation had no effect on 2 subjects with near normal initial DP values (Figure 5A). On average, DP decreased by up to 80% over the first 3 sessions. On the fourth session, 3 patients had normal DP values and were not subjected to the unidirectional rotation. For the last 2 sessions, only two of the patients showed initial abnormal DP values and were thus subjected to the unidirectional rotation. As such, the rehabilitation was effective in all cases and in most cases only required less than 3 sessions.

To investigate whether the effect of the rehabilitation was preserved between sessions, we compared the 70 min post-rehabilitation DP of each session with the initial DP of the next session (Figure 5B). As mentioned before, average initial DPs at the beginning of each session (i.e., before rehabilitation in that session) showed a decrease over time (Figure 4A). Interestingly, these average initial values were similar to after rehabilitation DPs from the previous session (Figure 5B, repeated measure ANOVA, *p* = 0.1), suggesting that the effect of rehabilitation was retained and did not substantially diminish between sessions. The seemingly larger differences between DPs (and larger variabilities) after session 4 are probably due to the longer times between sessions (i.e., twice weekly for the first 4 sessions and weekly for sessions 5 and 6).

### Effect of Unidirectional Rotational Rehabilitation on Subjective Symptoms

Six of the eight subjects that participated for more than 1 session in the long-term study reported on subjective changes in their symptoms over time. At the beginning of this study, two of the patients experienced only mild imbalance, while others had true vertigo or severe imbalance. However, these 2 patients showed other signs of vestibular dysfunction, such as falling toward one side in the dark. In all subjects, the sense of imbalance was aggravated by rapid head movements. All subjects also experienced associated autonomic symptoms during vertigo. Interestingly, the 2 subjects with the least response to the unidirectional rotation (Figure 5A) were the ones with a history of mild imbalance. All 6 subjects reported decrease in the intensity and frequency of dizziness/imbalance symptoms and felt more confident participating in social and daily activities.

## Discussion

### Unidirectional Rotational Rehabilitation Improves VOR Symmetry

The results of the present study show that training by a purely unilateral vestibular stimulation could decrease the asymmetry of the VOR response in patients with chronic vestibular dysfunction. We used a unidirectional rotational stimulation in the dark (i.e., without any visual stimulation) and showed that this could be an effective rehabilitation method for decreasing the DP of patients with chronic vestibular dysfunction. In most cases, the vestibular imbalance decreased by ~10 min after the rehabilitation was applied and the effect lasted for weeks. Although chronically elevated DPs are harder to change (39), we found that all subjects showed an improvement in their DPs with this rehabilitation method.

Repeated unidirectional rotations have previously been shown to result in habituation of vestibular responses and a decrease in VOR gain and time constant in different animals as well as humans (27–29, 38, 40). Indeed, with unidirectional stimulations, the gain of the stimulated side decreased over time while the opposite side showed no change in response (38). In our study, we observed the opposite effect: rotations toward the LR side resulted in an increase in their responses, while those of HR side slightly decreased. We believe that the difference between our results and those of previous studies is due to two factors. First, we used a purely unidirectional rotation and took special care to have a very slow deceleration in order to avoid any reversal of stimulation at the end of rotation. This is in contrast to previous studies where step stimuli were stopped abruptly and in fact the stimulus was considered to be the deceleration part of the movement (28, 40). As such, in previous studies subjects received vestibular stimulation in both directions, corresponding to the acceleration and deceleration parts of the movement. Second, our subjects were patients with asymmetric responses and some level of compensation. Previous studies during compensation have shown changes in properties of vestibular nuclei neurons (3, 4), inputs to the vestibular nuclei (6–8, 41), and commissural connections between the two sides (5). Because of the asymmetry and the above changes at the cellular, synaptic, and network levels, it is conceivable that repeated stimuli could have different effects (i.e., inducing a homeostatic change in the activity of vestibular nuclei to reach a new balance between the two sides) compared to normal conditions (i.e., habituation and a decrease in response).

It has been shown that the naturally occurring compensation could be improved further by specific goal-directed training exercises. Such rehabilitation exercises typically use the multisensory nature of vestibular compensation to further improve balance and gaze stability in patients. Animal studies have shown compensatory changes in the vestibular nuclei (VN) neuron responses, changes in extravestibular inputs (such as neck proprioception and efferent copy of neck motor command) to the VN (6–8) as well as changes at the peripheral level (2). Consistent with these studies, patients with vestibular dysfunction use compensation strategies that include changes in neck reflexes (42, 43), preprogramming of compensatory eye movements (44–46), and generation of multiple catch-up saccades (47–49).

Visual inputs play a major role in vestibular compensation so that when animals were kept in darkness for 4 days after unilateral lesion, they did not show improvement in spontaneous nystagmus, which was recovered once they were moved to a lighted area (50). Studies on animals with compensated asymmetric VOR responses after unilateral labyrinthectomy have shown that further general VOR adaptation could be attained to raise the gain of the VOR with repeated visual-vestibular interaction training. These studies used bidirectional rotations while viewing a patterned background (9) or unidirectional visual–vestibular training (i.e., providing retinal slip only during ipsilesional head rotations) (10) and showed that ipsilesional VOR gain could be selectively enhanced. The findings of these previous studies suggest that vestibular compensation does not reach its maximum capacity by spontaneous/natural recovery processes and the VOR gain could be further increased by visual–vestibular training after compensation.

The goal of rehabilitation exercises is to use visual and other extravestibular inputs as well as other balance cues to further compensate for the lack of vestibular inputs. Previous studies have shown a 70–80% improvement in patients using different rehabilitation protocols. One study found that 28% of patients showed complete resolution of symptoms within 1 year and 54% showed some degree of improvement (51). The unidirectional rotational stimulus that we used for rehabilitation was designed based on the theoretical and experimental observations showing that changes in the commissural pathway between the two vestibular nuclei (VN) contributes to vestibular compensation (5, 52–55). We expected that a purely vestibular stimulation would activate Hebbian plasticity mechanism in these pathways [i.e., cells that fire together wire together (56)]. During rotation, ipsilateral receptors are stimulated and contralateral ones are inhibited. At the VN level, the interaction between the two sides increases this imbalance. Type I excitatory neurons that are stimulated by ipsilateral inputs from the nerve, innervate contralateral type II inhibitory neurons, which project to and inhibit type I neurons on the same side. As such, unidirectional rotations toward the LR side will stimulate this weaker side and inhibit the stronger HR side, rebalancing the two sides. This suggests an increase in VOR gain for rotations in one direction and a decrease in VOR gain for rotations in the opposite direction. Indeed, recent studies have shown independent VOR gain adaptation to right and left rotations in normal humans (57–59). Initially, we were hoping to see a stronger effect on the LR and an improvement in the VOR response (gain) after rehabilitation. However, the VOR response became more symmetric (i.e., lower DPs) due to a non-significant decrease in VOR response to HR rotation and a non-significant increase in LR responses. It is possible that the increase in LR response was diminished by a concomitant habituation to repeated rotations, as suggested by previous studies in normal subject [e.g., (38)].

Mean values of DP during the 6 sessions show reductions and even reversals, demonstrating the effect of the rehabilitation to reduce the vestibular imbalance. Most of the changes were observed in the first session with about 80% decrease in DP measured 70 min after the rehabilitation. This is similar to the results of Ushio et al. (10), where a significant change was observed immediately after their unidirectional visual–vestibular adaptation paradigm in animals after unilateral labyrinthectomy. We observed that in most cases even 2–3 such unidirectional rotations (albeit in the dark) would result in normal DPs. Note that this normalization of DP is partly mediated by an increase in the response of LR side, thus improving the overall vestibular function (Figures 2A, 3B, 4B,C). Furthermore, the effect of rehabilitation did not seem to be dependent on the initial DP value. From the 6 subjects, 4 were sensitive and showed a decrease of >100% (i.e., a change in the direction of DP) by the last session. Two other patients showed very little change (i.e., ~15%) over the 6 sessions, yet had initial DP values close to those of 2 of the patients that were sensitive to the rehabilitation.

We observed a retention of the rehabilitation effect for days to weeks in most patients. This is in contrast to results of Ushio et al. (10), where the unidirectional visual–vestibular training effect was preserved only for faster movements (i.e., during the acceleration period of their velocity trapezoid test rotation) 3 days after the last session. This apparent discrepancy could be due to multiple factors. The most important difference is the adaptation pathways used in the two studies. This previous study used visual-vestibular adaptation that is mediated through the cerebellum and floccular target neurons in the VN as part of the “modifiable VOR pathway” (60–63). We used rotation in the dark, which should affect all VN neurons regardless of their type. Furthermore, the dynamics of our stimulus were very different from that used by Ushio et al. (10). In our study, unidirectional rotations reached a peak velocity of 320°/s, which is higher than the 150°/s used in the previous study. For our purposes, it was critical to have a slow deceleration (10°/s^2^) in order to avoid stimulation in the opposite direction when stopping the rotation. This is very different from the 1,000°/s^2^ acceleration/deceleration used by the previous study. It was suggested that the training provided by the previous study most likely affected the irregular/phasic pathway (10). In contrast, we believe that the present study most likely affected the tonic pathway, with stronger long-term effects when tested by slow sinusoidal rotations. Whether we also affected the phasic pathway (i.e., response to faster head movements) was not tested due to the limitation of ENG (rather than VNG) testing and safety issues of rotation of human subjects by the chair at high frequencies and velocities. Using the head impulse test with VNGs could address this point more clearly in future studies. Finally, there could be species differences between humans (present study) and monkeys used in the previous study.

While both the visual–vestibular training (10) and the unidirectional rotation introduced in our study show similar efficiency in increasing the vestibular compensation, because of different pathways involved, the two methods could have different clinical applications. The visual–vestibular training functions through the adaptation pathway and as such, is not appropriate for patients with damage to areas such as the cerebellum. In contrast, our unidirectional rotation in the dark most likely affects neurons in the vestibular nuclei and the commissural pathway (rather than the cerebellum). Consistent with this notion, previous studies on habituation of responses to repeated rotations in normal subjects have concluded that changes occur mainly in the velocity storage, which is part of the vestibular nuclei (27). The unidirectional rotations described in the present study also have the benefit of being simpler to perform and require simpler equipment, with no visual stimulation.

### Effect of Unidirectional Rotation on Subjective Symptoms

To evaluate the subjective improvement of symptoms, we used a simple questionnaire. The patients were required to report the frequency and intensity of symptoms that they mentioned on the first session, as well as any new symptoms developed during the study. Surprisingly, although previous studies have shown that DP is a good measure of the degree of compensation in the vestibular system (34, 64, 65), we found a discrepancy between improvement in DP values and subjective improvement following vestibular rehabilitation. Only 6 out of 16 patients reported subjective improvement in symptoms during the rehabilitation program. However, it is important to note that the rehabilitation and rotation tests did not result in aggravation of any of the symptoms.

Previous studies have shown that training and adaptation in one direction of movement does not necessarily transfer to other types of movements (66–68). As a result, patients with major problems in the horizontal rotation response would have benefited the most from the present rehabilitation. In the present study, we only measured the function of the horizontal VOR responses. Future studies for measurement of responses to roll, pitch, or linear movements are required to directly study whether the effect of this rehabilitation in the horizontal plane could transfer to any of these other directions of movement.

It should be noted that in the present study, rather than the available standardized questionnaires, we used a simple form for following up the symptoms in our subjects. Standard questionnaires are detailed and long and while they are excellent for initial careful validation of symptoms in vestibular patients, they are cumbersome for using multiple times over a short period of time. We used a short form between visits to simply verify any change in the progression of patients' imbalance as they conceived it. However, it should be noted that since our questionnaire was not validated by a large number of patients, response variability could be higher and potentially be a source of discrepancy between subjective and objective results. Future studies that include quantification methods (such as Likert scale) on standardized tests are required to further evaluate the subjective effect of this rehabilitation and a comparison to other methods such as the vestibular-visual training.

In general, one of the shortcomings of subjective measures is that their value is reduced due to their intrinsic variability. The observed discrepancy between the subjective and objective improvement could be related to psychological factors (e.g., fear of movement) and personal characteristics (e.g., age, sex, income, educational level, comorbidities, and motivation) (69, 70). It has been shown that the fear of recurrences is at the root of the psychosocial disabilities associated with vertigo and there is a strong relationship between the severity of such disability and the accompanying somatic anxiety (71–73). Since 2014, new criteria have been set to diagnose psychological consequences or causes of chronic dizziness as “persistent postural-perceptual dizziness” (PPPD), which includes anxiety, panic attacks, and depression (74). It has been shown that PPPD caused by vestibular problems can be decreased by vestibular rehabilitation. On the other hand, it has also been demonstrated that there is no correlation between the frequency of symptoms and the degree of disability in patients, as some patients who experience permanent instability may be significantly less affected in their daily lives than patients who suffer from dizziness less often (75). Future studies with larger number of patients over a longer period of time and by taking into account recent criteria for identification of different etiologies and psychological factors as identified by the International Classification of Vestibular Disorders (76) in individual subjects are required to more accurately investigate any direct relationship between subjective and objective measures of improvement after rehabilitation.

## Author Contributions

NR conceptualized the original idea and hypothesis behind the unidirectional rotational rehabilitation. NR and SGS designed the study, analyzed the data, and wrote the manuscript. NGS designed the study, performed the experiments, analyzed the data, and wrote the manuscript. BS designed the study and performed the experiments.

### Conflict of Interest Statement

The authors declare that the research was conducted in the absence of any commercial or financial relationships that could be construed as a potential conflict of interest.
